# Maize Responses Challenged by Drought, Elevated Daytime Temperature and Arthropod Herbivory Stresses: A Physiological, Biochemical and Molecular View

**DOI:** 10.3389/fpls.2021.702841

**Published:** 2021-07-21

**Authors:** Cristhian Camilo Chávez-Arias, Gustavo Adolfo Ligarreto-Moreno, Augusto Ramírez-Godoy, Hermann Restrepo-Díaz

**Affiliations:** Universidad Nacional de Colombia, Sede Bogotá, Facultad de Ciencias Agrarias, Departamento de Agronomía, Bogotá, Colombia

**Keywords:** *Zea mays* L, stress combination, climate change, drought, high temperature, herbivorous arthropods

## Abstract

Maize (*Zea mays* L.) is one of the main cereals grown around the world. It is used for human and animal nutrition and also as biofuel. However, as a direct consequence of global climate change, increased abiotic and biotic stress events have been reported in different regions of the world, which have become a threat to world maize yields. Drought and heat are environmental stresses that influence the growth, development, and yield processes of maize crops. Plants have developed dynamic responses at the physiological, biochemical, and molecular levels that allow them to escape, avoid and/or tolerate unfavorable environmental conditions. Arthropod herbivory can generate resistance or tolerance responses in plants that are associated with inducible and constitutive defenses. Increases in the frequency and severity of abiotic stress events (drought and heat), as a consequence of climate change, can generate critical variations in plant-insect interactions. However, the behavior of herbivorous arthropods under drought scenarios is not well understood, and this kind of stress may have some positive and negative effects on arthropod populations. The simultaneous appearance of different environmental stresses and biotic factors results in very complex plant responses. In this review, recent information is provided on the physiological, biochemical, and molecular responses of plants to the combination of drought, heat stress, and the effect on some arthropod pests of interest in the maize crop.

## Introduction

Plants are exposed to a wide range of abiotic and biotic stresses that induce a disruption in plant metabolism (Atkinson and Urwin, 2012; Zhang and Sonnewald, 2017; Vemanna et al., 2019) which, in turn, leads to a reduction in growth and yield (Rejeb et al., 2014; Pandey et al., 2017). These types of stresses are common in many agricultural areas around the world, and represent one of the main threats of interest to crop productivity worldwide (Sewelam et al., 2014; Chojak–Koźniewska et al., 2018). Drought (water deficit in the soil or plant water scarcity), heat (elevated air temperature), cold, salinity, high light intensity, high CO_2_ concentrations, weeds, diseases, and pests are some of the abiotic and biotic stresses that have been studied the most (Suzuki et al., 2014; Pandey et al., 2017). The majority of the studies on the different types of stresses have been carried out individually and under controlled conditions, whereas field studies generally show the effect of the combination of such factors (Mittler, 2006; Suzuki, 2016a). The combination of two or more types of stress is common in many agricultural areas of the world, causing considerable reductions in crop yields (Table 1; Suzuki et al., 2014; Ahmad et al., 2019).

**TABLE 1:** Impact of the different combinations of environmental stresses on the plant.

**Combination**	**Stress combinations**	**References**
Negative combination	Drought + heat	Zandalinas et al., 2018; Cohen et al., 2021
	Drought + salinity	Sahin et al., 2018
	Drought + chilling	Hussain et al., 2018
	Drought + UV	Rodríguez–Calzada et al., 2019
	Drought + pathogen	Tani et al., 2018
	Drought + pets	Shehzad et al., 2021
	Drought + nutrients	Mittler and Blumwald, 2010
	Drought + high light	Carvalho et al., 2016
	Heat + ozone	Pliūra et al., 2019
	Heat + salinity	Suzuki et al., 2016b
	Heat + pathogen	Pandey et al., 2017
	Heat + pets	Nguyen et al., 2016
	Heat + UV	Mittler and Blumwald, 2010
	Heat + high light	Carvalho et al., 2016
	Salinity + ozone	Peykanpour et al., 2016
	Salinity + pathogen	Bai et al., 2018
	Salinity + nutrients	Suzuki et al., 2014
	Chilling + high light	Terada et al., 2018
	Pathogen + nutrients	Mittler and Blumwald, 2010
Positive combination	Drought ozone	Mittler and Blumwald, 2010; Suzuki et al., 2014
	Drought + high CO_2_	van der Kooi et al., 2016
	Salinity + high CO_2_	Sgherri et al., 2017
	Ozone + pathogen	Mittler and Blumwald, 2010
	Ozone + high CO_2_	Ainsworth, 2008
	Pathogen + UV	Mittler and Blumwald, 2010; Suzuki et al., 2014
	High CO_2_ + high light	Mittler and Blumwald, 2010; Suzuki et al., 2014

In recent decades, global warming due to climate change has been accelerated by the higher concentration of CO_2_ in the atmosphere. This phenomenon is generating an increase in the average temperature, alterations of precipitation patterns, and reduction of arable land and water resources in agricultural areas of the world (Ahmad et al., 2019; Dong et al., 2020). Additionally, complex combinations of abiotic stresses such as drought and salinity, salinity and heat, and drought and extreme temperatures due to climate change are expected to occur in many agricultural areas across the globe (Table 1; Suzuki et al., 2014; Wijewardene et al., 2020). On the other hand, the behavior of some arthropod pests may also be influenced by climate changes and abiotic stresses (Nguyen et al., 2016; Bonnet et al., 2017). Copolovici et al. (2014) reported that changes in plant metabolic processes driven by abiotic stress can affect the response of plants to attack generated by arthropod pest herbivory.

As a result of climate change, the most limiting abiotic factors for crop productivity and food security are drought and heat (Fahad et al., 2017; Hussain et al., 2019). Studies on the effect of drought and heat on plant cultivation have been widely documented (Zhou et al., 2017; Guo et al., 2018; Haworth et al., 2018; Osmolovskaya et al., 2018). However, research on the combination of heat stress and drought is not common even though these two types of abiotic stresses usually appear simultaneously under field conditions and generate harmful effects on crop growth and productivity (Table 1; Suzuki et al., 2014; Raja et al., 2020). The combination of water deficit (drought) and heat (increases in average air temperature above the optimum) stresses can alter physiological, biochemical, and molecular processes in plants (Prasad et al., 2008; Lamaoui et al., 2018).

In general, the combination of drought and high daytime temperatures reduces the photosynthetic efficiency, stomatal conductance, leaf area, water use efficiency (WUE), and yield of plants (Sattar et al., 2020). The effect of the combination between drought and heat has been reported in some crops of agricultural interest such as lentil (*Lens culinaris* Medikus; Sehgal et al., 2017), chickpea (*Cicer arietinum* L.; Awasthi et al., 2014), tomato (*Solanum lycopersicum* L.; Zhou et al., 2017), wheat (*Triticum aestivum* L.; Liu et al., 2018), *Jatropha curcas* L. (Silva et al., 2010), and citrus trees (Zandalinas et al., 2017). These reports show that the combined effect of drought and heat on plant growth and productivity is more severe than the individual effects of these factors (Awasthi et al., 2014; Urban et al., 2018).

Plants are subject to the herbivory of a wide range of phytophagous arthropods during their growth and development; therefore, this factor is one of the main types of biotic stress affecting crop growth (Zhou et al., 2015; Bonnet et al., 2017). Several studies indicate that environmental stresses, such as drought or high temperatures, can make plants more susceptible to arthropod feeding and attack because of a drop in plant defense mechanism (DeLucia et al., 2012; Gutbrodt et al., 2012; Weldegergis et al., 2015; Havko et al., 2020; Table 1). The combination of the effects of drought or high temperatures and the presence of herbivorous arthropods has been investigated in plants such as mountain Avens (*Dryas octopetala*; Birkemoe et al., 2016), tomato (Havko et al., 2020), arabidopsis (*Arabidopsis thaliana* L.; Davila Olivas et al., 2016), apple (*Malus x domestica* Borkh.; Gutbrodt et al., 2012), and bittersweet nightshade (*Solanum dulcamara* L.; Nguyen et al., 2016).

Maize (*Zea mays* L.), along with wheat and rice, is one of the main staple foods in the world with a global production of more than 1 × 10^9^ *t* since 2013 (Noman et al., 2015; Zampieri et al., 2019). Maize is grown for various purposes, such as human consumption, animal feed, forage production, and renewable energy (bioenergy; Aslam et al., 2015; Ai and Jane, 2016). In many regions of the world, maize is commonly grown in semi-arid environments characterized by low water availability and high daytime temperatures, two environmental factors that usually occur simultaneously in the field (Hu et al., 2015; Zhao et al., 2016). Maize crops are extremely sensitive to heat and drought stresses (Zhao et al., 2016). According to Hussain et al. (2019), world maize yield and production are projected to decline by 15–20% per year due to heat and drought conditions, with these two factors becoming major threats to this crop. On the other hand, a reduction in maize yield of 6–19% caused by arthropods and other herbivores has also been reported (Block et al., 2019). *Rachiplusia nu* (Guennée; Lepidoptera: Noctuidae; Russo et al., 2019), fall armyworm [*Spodoptera frugiperda* (JE Smith; Lepidoptera: Noctuidae)] (Pannuti et al., 2016), black cutworm [*Agrotis ípsilon* (Hufnagel; Lepidoptera: Noctuidae)] (Yan et al., 2020), cotton bollworm [*Helicoverpa armígera* (Hübner; Lepidoptera: Noctuidae)] (Gomes et al., 2017), corn earworm [*Helicoverpa zea* (Boddie; Lepidoptera: Noctuidae)], and thrips [*Frankliniella williamsi* (Thysanoptera: Thripidae)] (Manandhar and Wright, 2016) are some of the main pests reported for this crop.

The results of studies on the responses and adaptations of maize plants exposed to abiotic and biotic stresses have been well documented separately (Zhao et al., 2016; Block et al., 2019). However, the information available remains limited to physiological responses of maize plants exposed to the combination of multiple abiotic and biotic stress factors, such as drought, high temperature and arthropod pest herbivory. Therefore, more studies are necessary to continue understanding the effects of these multiple combinations. Due to the complexity of the combinations of drought, heat, and arthropod pests, this review aimed to report the effects of the combination of abiotic (drought and heat) and biotic (herbivory) stresses on the physiological, biochemical, and molecular mechanisms of maize plants.

## Physiological Responses to the Combination of Heat and Drought Stress in Maize

Plant responses to the combination of drought and heat depend on the intensity, frequency, and duration of the interaction between both stresses, as well as the stage of phenological development of the crop (Prasad et al., 2008; Fahad et al., 2017). The individual and combined effects of drought and heat on the physiological response of maize plants have been widely documented, which are summarized in Table 2. The combined effects of these two environmental stresses have a greater negative impact on plant growth compared to the effect of each individual factor (Killi et al., 2017; Hussain et al., 2019).

**TABLE 2:** Summary of the impact of heat and drought stress (individual or in combination) on the physiological responses of maize (*Zea mays* L.) plants.

**Stress**	**Effect of stress on the plant**	**References**
Drought	Increase in flowering days and days of maturity, decrease in the number of leaves, loss of root architecture, and reduction in yield in susceptible and tolerant lines.	Sah et al., 2020
	Reduction in growth, fresh and dry biomass, and photosynthetic pigments.	Noman et al., 2015
	Reduction in water status, photosynthetic pigments, and yield.	Nawaz et al., 2016
	Reduction in water status, growth, gas exchange parameters (photosynthesis, stomatal conductance, transpiration), chlorophyll content, and photochemical efficiency of PSII (F_v_/F_m_).	Ye et al., 2016, Li et al., 2018
Heat	Inhibition of seed germination.	Zhou et al., 2018
	Reduction in plant height, foliar area, and dry matter accumulation. Decrease in F_v_/F_m_ and chlorophyll content.	Sunoj et al., 2016
	Reduction in growth and yield parameters. Decrease in gas exchange parameters (photosynthesis, stomatal conductance, transpiration), chlorophyll content, and photochemical efficiency of PSII (F_v_/F_m_).	Mathur et al., 2018
Drought and heat	Reduction in yield.	Tesfaye et al., 2018
	Decrease in yield, plant height, and anthesis and silking dates.	Obata et al., 2015
	Reduction in growth parameters (height, stem diameter, leaf area, fresh and dry weight), yield, water status, and nutrient content in the plant. Decrease in chlorophyll content and gas exchange parameters such as photosynthesis, stomatal conductance, and transpiration.	Hussain et al., 2019
	Reduction in fresh and dry weights and plant transpiration. Increase in leaf temperature.	Ayub et al., 2020
	Decrease in yield and increase in days from anthesis to silking.	Cairns et al., 2013

**FIGURE 1:**
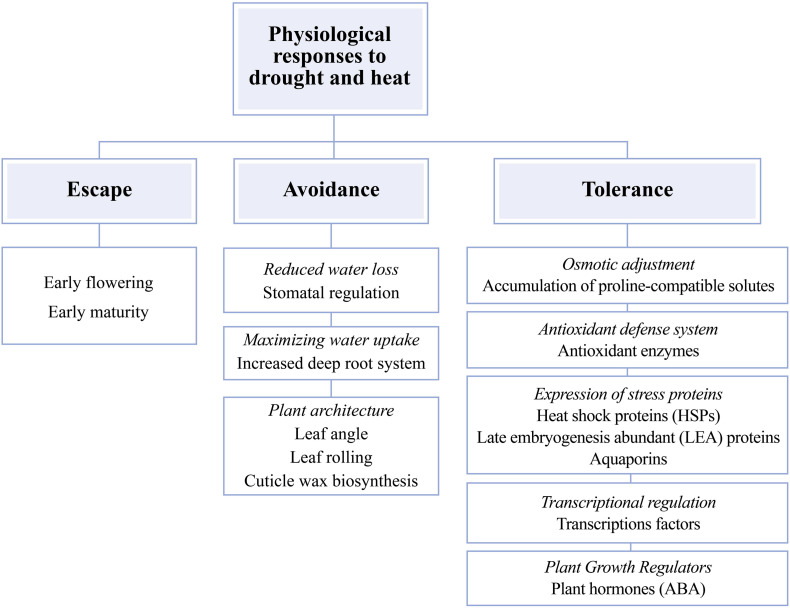
Mechanisms of morphological, physiological, biochemical, and molecular responses to the combination of drought and heat in maize (*Zea mays* L.) plants. Adapted from Aslam et al. (2015) and Tiwari and Yadav (2019). Words in italics represent physiological, biochemical and molecular plant mechanisms, non-italicized words represent biological processes affected by abiotic stresses.

The physiological responses of maize plants to drought and heat can be classified into three different mechanisms: escape, avoidance, and tolerance (Figure 1). In the escape mechanism, the plant tries to complete the reproductive stage before the stress became more severe (Aslam et al., 2015; Khan et al., 2019). Avoidance mechanisms are mainly morphological and physiological changes that allow reducing exposure to the combination of drought and heat (Figure 1; Zhang et al., 2016; Lamaoui et al., 2018). Avoidance of drought or heat stress damage can be achieved by increasing root system to maintain water uptake (Aslam et al., 2015). Also, the effects of any of the two stresses can be avoided by changing plant architecture. Some of these changes may include a lower leaf angle, reduced leaf rolling, compact tassel, and efficient cuticle wax biosynthesis to reduce direct sunlight exposure and evapotranspiration rates (Aslam et al., 2015; Tiwari and Yadav, 2019), and reduced leaf stomatal number and conductance to avoid water losses to keep water status (Figure 1; Aslam et al., 2015; Lamaoui et al., 2018). Finally, tolerance to the combination of heat and drought stress is the ability to keep growth and development through cellular and biochemical modifications. These changes include the accumulation of compatible osmolytes [proline, glycine betaine, soluble sugars, and inorganic ions (K^+^, Na^+^, Ca^2+^, Mg^2+^, Cl^–^, and NO_3_^–^)] to support plant water status through osmotic adjustment (Blum, 2017; Lamaoui et al., 2018) and the activation of the enzymatic and non-enzymatic antioxidant system [superoxide dismutase (SOD), catalase (CAT), peroxidase (POD), and ascorbate peroxidase (APX)] (Aslam et al., 2015; Hussain et al., 2019) and growth regulators (plant hormones) such as abscisic acid (ABA; Aslam et al., 2015; Lamaoui et al., 2018). Other modifications also include the activation of transcription factors (TFs) that regulate expression levels of genes sensitive to the combination of drought and high temperatures (Lamaoui et al., 2018) and the overexpression of stress proteins such as heat shock proteins (HSP), late embryogenesis abundant (LEA) proteins, and aquaporins (that intervene in the movement of water under stress; Figure 1; Khan et al., 2019; Tiwari and Yadav, 2019).

In maize, the combination of drought and heat reduces the photosynthetic rate, stomatal conductance, leaf area, and WUE (Sehgal et al., 2017). Plant water status parameters such as relative water content (RWC), leaf water potential (Ψ_h_), osmotic potential (Ψ_*s*_), and turgor potential (Ψ_*t*_) decrease progressively under drought and exposure to high temperatures (>35°; Shah and Paulsen, 2003). Hussain et al. (2019) reported a greater reduction in RWC values in two maize hybrids subjected to the combination of drought and heat, compared to the individual effects of each abiotic stress. Under water shortage, root hydraulic conductivity can be reduced to avoid water losses in the plant. This effect can be more severe under heat stress, causing roots damage (Lamaoui et al., 2018).

Photosynthesis in C4 plants is more sensitive to drought periods due to stomatal closure and the reduction in the activity of photosynthetic enzymes compared to C3 plants (Ghannoum, 2009; Lipiec et al., 2013). Under thermal stress, photosynthesis in C4 plants shows a greater tolerance than in plants with C3 metabolism, associated with the accumulation of oxaloacetic acid within the bundle sheath cells. This process effectively concentrates CO_2_ at the carboxylation site of Rubisco, suppressing photorespiration (Killi et al., 2017, 2020). Water scarcity is known to affect the electron transport chain through inhibition of D1 synthesis and damages to the oxygen-evolving complex in the PSII and in the reaction centers of both PSII and PSI (Dalal and Tripathy, 2018; Wang et al., 2018). Also, water deficit reduces the abundance of proteins involved in Calvin-Benson-Bassham cycle, such as ribulose 1,5-bisphosphate carboxylase (RuBisCO), Fructose 1,6-bisphosphate aldolase, triosephosphate isomerase, and glyceraldehyde phosphate dehydrogenase A (Burgess and Huang, 2016; Dalal and Tripathy, 2018). On the order hand, heat stress affects photosynthesis since it could cause a lesion on the thylakoid membranes (Ivanov et al., 2017), directly damage the photosynthetic apparatus, such as the PSI and PSII reaction centers, and in the cytochrome b6f (Cytb6f) complex, and decrease the activity of Rubisco (Hu et al., 2020). Perdomo et al. (2017) found a decrease in the Rubisco activity and the electron transport rate in maize plants subject to drought and high daytime temperatures evaluated individually.

Photosynthesis rate is extremely sensitive to the combination of heat and drought stresses (Perdomo et al., 2015). This sensitivity can be a consequence of the stomatal closure induced by the combination of these stresses, but it can also be attributed to non-stomatal limitations such as decreased leaf expansion, lower content of photosynthetic pigments, and inadequate functioning of the photosynthetic machinery (Fahad et al., 2017; Lamaoui et al., 2018). Hussain et al. (2019) observed a decrease in the photosynthetic rate of two maize hybrids; this drop is associated with the reduction in the total chlorophyll content and stomatal conductance values under the combination of drought and heat. Under high daytime temperatures, C4 plants, such as maize, open stomata to cool leaves by increasing the transpiration rate (Perdomo et al., 2015). However, the combination with drought generates a reduction in the values of stomatal conductance and transpiration in the same species (Hussain et al., 2019; Sabagh et al., 2020).

Regarding the phytochemical machinery, the activity of PSII and its maximum quantum efficiency (F_v_/F_m_) decrease after exposure to the combination of drought and heat (Alhaithloul, 2019). Killi et al. (2017) recorded a reduction in the F_v_/F_m_ ratio and actual quantum yield of PSII (ΦPSII), and an increase in non-photochemical quenching in two maize varieties that are susceptible and tolerant to drought when subjected to the combination of high temperatures and drought stress. These responses may also be associated with the disturbance of the integrity and fluidity of thylakoid membranes, and inhibition of electron transfer due to oxidative stress damage in the plant (Tian et al., 2013; Mathur et al., 2014; Talaat, 2020). Chlorophyll *a* fluorescence (an indicator of PSII performance) has been used as a quantitative measure of the impact of drought and heat on the functionality of thylakoid membranes in crops (Sehgal et al., 2017; Killi et al., 2020).

The reproductive stage is more sensitive to the combination of drought and heat than the vegetative stages (Obata et al., 2015; Sehgal et al., 2017). The reproductive processes most susceptible to the combination of heat and drought stress are pollen and stigma viability, pollen tube growth, early embryo development, flowering and seed filling, and number of kernels (Zandalinas et al., 2017; Lamaoui et al., 2018; Sehgal et al., 2019). Shah and Paulsen (2003) reported that stress during the reproductive phase also induces the abortion of kernels, possibly by decreasing the supply of carbohydrates and negatively affecting plant yield. In maize plants subjected to heat stress and drought, yield parameters such as kernels⋅ear^–1^, 100-kernel weight, kernel yield⋅plant^–1^, and harvest index were significantly reduced (Hussain et al., 2019).

## Biochemical and Molecular Responses to the Combination of Heat and Drought Stress in Maize

The genes expression related to water channel proteins and ion transporters is a combined strategy to reduce the impact generated by environmental stress factors (Afzal et al., 2016; Kido et al., 2019). These strategies also rely on genes related to the protection of membranes and essential proteins such as chaperones, HSP and osmoprotectant osmolytes (Bandyopadhyay et al., 2019; Kido et al., 2019). The accumulation of compatible osmolytes in plants subjected to stress due to drought, heat and their combination has been linked to the protection of protein structures and the stabilization of cell membranes to restore homeostasis (Lamaoui et al., 2018). The main osmoprotectants are those derived from polyamines, amino acids, soluble carbohydrates (e.g., glucose), betaines (e.g., glycine betaine), and sugar alcohols (Lamaoui et al., 2018; Kido et al., 2019). Proline is one of the most studied osmolytes under water deficit and heat stresses periods. It is a proteinogenic amino acid involved as an osmoprotectant of membranes and proteins. Proline also generates scavenging of reactive oxygen species (ROS) and is considered a compatible osmolyte for the osmotic adjustment of cells (Zouari et al., 2019; Zulfiqar et al., 2020). Ayub et al. (2020) reported the accumulation of proline and total soluble sugars in four maize hybrids subjected to the combination of drought and high temperatures. The proline synthesis and accumulation have been used as a tolerance trait to abiotic stresses (Lamaoui et al., 2018).

Another tolerance mechanism that plants have to adapt to heat and drought stresses is the induction of antioxidant enzymes such as CAT, glutathione peroxidase, total superoxide dismutase (T-SOD), POD, APX, and glutathione reductase (GR). Additionally, plants induce non-enzymatic antioxidants such as glutathione (GSH) and ascorbate to control ROS concentration (Zandalinas et al., 2017). In maize, lower concentrations of ROS [as superoxide anion (O_2_^–^), hydrogen peroxide (H_2_O_2_), and hydroxyl free radical (OH^–^)] and malondialdehyde (as a by-product of lipid peroxidation) have been reported with the increase in the activity of enzymatic antioxidants such as T-SOD and POD, and non-enzymatic antioxidants such as GSH in two maize materials under the combination of drought and heat (Hussain et al., 2019).

Plant hormones are also involved in the regulation of tolerance to environmental stresses (drought and heat; Lamaoui et al., 2018). ABA is the most important plant hormone that intervenes in the regulation of plant acclimation to drought and heat (Zandalinas et al., 2017). The increase in ABA accumulation is also associated with tolerance to the combination of heat and drought since it regulates stomatal opening and closure, and activates antioxidant defense systems. It also stimulates the production of dehydrins and LEA proteins that participate in osmotic adjustment and other plant protection mechanisms (Haider et al., 2018; Lamaoui et al., 2018; Zhang et al., 2019). However, the previous responses mediated by the endogenous accumulation of ABA are associated with plant tolerance to the abiotic stress factor studied individually and not in combination (Hu et al., 2010). In maize, an endogenous accumulation of ABA has been reported in two cultivars (tolerant and sensitive to drought) after 12 days of exposure to drought (Zhang et al., 2012). Also, Cheikh and Jones (1994) reported an increase in ABA levels in the female inflorescence (ear) of maize plants exposed to a temperature of 35°C for 8 days. Plant hormones such as cytokinins and auxins play an important role in responses to abiotic stress (Bielach et al., 2017). These hormones are related to the stimulation of cell division and the control of plant growth and development. Additionally, their ability to crosstalk promotes adaptive responses to different types of stress (Dobra et al., 2010; Bielach et al., 2017). For example, Bedada et al. (2016) reported an increase in endogenous levels of cytokinins through the expression of *IPT* genes that encode the activity of the enzyme isopentenyltransferase. This enzyme catalyzes the synthesis of cytokinins, which may be related to an increase in tolerance to water deficit in maize plants.

Cell molecular events can be affected by environmental stresses (Aslam et al., 2015; Guo et al., 2016). Plant survival strategies under drought and high daytime temperatures periods occur through the modification of gene expression, which results in the generation of certain proteins known as stress proteins (Aslam et al., 2015; Shafqat et al., 2021). Water channel proteins such as aquaporins (AQP), LEA, and HSP are some of the proteins that play an important role in plant tolerance to stress caused by drought, heat, and their combination (Priya et al., 2019). AQP are integral proteins found in tonoplasts, plasma membranes, and other intracellular membranes that are abundantly expressed in roots (Shafqat et al., 2021). Under water shortage, they preserve cell homeostasis by preventing water loss and increase membrane permeability. AQP also play a role in maintaining the WUE and signaling at the whole plant level by interacting with ROS in response to external signals (Aslam et al., 2015; Priya et al., 2019). Tonoplast intrinsic proteins, membrane intrinsic proteins, nodulin-like proteins (NIP), and plasma membrane intrinsic proteins (PIP) are subfamilies of AQP that promote water transport and tolerance to water and heat stress in maize plants (Aslam et al., 2015; Hu et al., 2015). Hu et al. (2015) found that aquaporin PIP2-7 and the integral membrane protein of NIP type showed a positive regulation under the combination of drought and heat in maize plants.

Under combined stresses (water deficit and high daytime temperatures), it is possible for plants to induce the transcription of proteins related to cell protection against dehydration (Nagaraju et al., 2019). LEA are some of the proteins involved in resistance to environmental stresses, mainly drought, as they accumulate in stressed tissues (Magwanga et al., 2018). They can also act as chaperones, preventing dehydration in some developmental stages susceptible to water limitation such as seed, pollen grain, shoot, and root development under drought (Pedrosa et al., 2015; Kaur and Asthir, 2017). Li and Cao (2016) identified a total of 32 genes related to LEA proteins in maize plants subjected to drought stress.

The expression of HSP is an adaptation strategy to combined stresses (drought and heat), and their accumulation correlates with stress tolerance in plants (Grigorova et al., 2011; Lamaoui et al., 2018). HSP act as molecular chaperones, preventing the aggregation of denatured proteins, stabilizing membrane proteins, facilitating protein folding, and allowing the renewal of normal cellular and physiological activities which contribute to a higher level of tolerance to stress (Priya et al., 2019; Khan and Shahwar, 2020). In maize plants, two types of HSP families, HSP90 and HSP100, have been reported in response to drought stress. These two families are located in the cytosol, nucleus and endoplasmic reticulum of the cell and have role in the translocation of proteins, regulation of steroid hormone receptors, and folding of proteins (Khan and Shahwar, 2020). Hu et al. (2010) found a strong correlation between the accumulation of proteins sHSP17.2, sHSP17.4, and sHSP26 and tolerance of the whole plant to the combination of drought and heat. Hussain et al. (2019) also reported a greater accumulation of HSP in two maize hybrids subjected to the combination of drought and high temperatures.

Drought and heat also generate changes in the cell plasma membrane, damaging the permeability of this cell structure (ElBasyoni et al., 2017). Damage to cell membrane permeability due to these abiotic stress conditions may be caused by membrane protein denaturation and enzyme inactivation, and results in the cell being unable to maintain its organic composition (ElBasyoni et al., 2017; Nijabat et al., 2020). The alteration in the permeability and integrity of the cell membrane generates a lower ion flow and higher electrolyte leakage. It also causes imbalances in the RWC, increases in the production of toxic compounds, and alterations of homeostasis. These changes inhibit cell viability, which is reflected in lower growth and development of plants exposed to drought and high daytime temperatures (Alhaithloul, 2019; Nijabat et al., 2020). Electrolyte leakage is a technique that has been used to evaluate cell membrane stability as a mechanism of tolerance to heat and drought stresses (Bajji et al., 2002; Ilík et al., 2018). Increased cell membrane damage expressed as higher electrolyte leakage has been reported in maize plants subjected to drought (Chen et al., 2010) and high daytime temperatures (Takele, 2010).

The TFs responsive to stress are the main cellular mechanisms related to plant tolerance to environmental adverse conditions (Zandalinas et al., 2017). TFs have been isolated from different genes related to abiotic stress and can regulate various complex pathways by modifying metabolite fluxes to improve stress tolerance (Aslam et al., 2015; Lamaoui et al., 2018). The main families of stress-related TFs include DREB, ERF, WRKY, MYB, bHLH, bZIP, DOF, and NAC (Lamaoui et al., 2018). Shi et al. (2017) observed that overexpression of the *ZmARGOS1* gene in maize plants improved tolerance to drought. The overexpression of *OsMYB55* in maize plants has led to an increase in the tolerance to the combination of drought and heat, which resulted in better growth and yield of plants under this environmental interaction (Casaretto et al., 2016).

## Maize Plant Responses to Arthropod Herbivory

Plants and arthropods have coevolved for more than 350 million years (Zhu et al., 2014). Arthropods have evolved to be able to locate host plants and oviposit using physical and chemical cues from those plants (Wu and Baldwin, 2010). Arthropod herbivory is a major biotic stress under natural conditions. For this reason, plants have developed different types of defenses, such as constitutive and inducible defenses, to resist or reduce the effects of arthropod attacks (Bonnet et al., 2017).

The plant has two defense strategies against herbivory attack: (i) resistance, which occurs when the plant prevents arthropod herbivores from feeding, and (ii) tolerance, which occurs when plant traits reduce the negative effect of herbivore damage (Gatehouse, 2002; Mitchell et al., 2016). One of the traits or responses related to crop resistance to arthropod herbivory is the chemical deterrence of pest settling and feeding (Mitchell et al., 2016). The deterrence of herbivore feeding is caused by the mixture of volatile and non-volatile compounds (War et al., 2012). Herbivore induced plant volatiles play an important role in defending the plant against the attack of arthropod pests by attracting the natural enemies of arthropod herbivores and acting as deterrents of their feeding and/or oviposition (War et al., 2012; Mitchell et al., 2016). The most common volatiles are green leaf volatiles (aldehydes, alcohols, and esters), aromatic compounds, and terpenes (Qi et al., 2018). In maize, aromatic compounds such as indole and methyl salicylate have been detected reducing the consumption and oviposition of arthropod herbivores (Qi et al., 2018). Ortiz–Carreon et al. (2019) identified the emission of three types of volatiles (α-pinene, α-longipinene, and α-copaene) after the damage of *S. frugiperda* larvae. Additionally, the emissions of these compounds attracted females of the endoparasitoid *Chelonus insularis* Cresson (Hymenoptera: Braconidae).

Structural traits of the plant such as trichomes, spines, waxy cuticles, or sclerophylly, can play the role of physical barriers to prevent the attachment, feeding and/or oviposition of arthropod pests (Santamaria et al., 2013). Trichome density and plant cuticle are the most studied traits in crop protection (Mitchell et al., 2016). Trichomes can prevent the attachment of arthropod pests and decrease their movement in crops (Andama et al., 2020). Trichomes also play a role in the interaction between plants and abiotic stresses, reducing heat loss from plants and increasing plant resistance to drought damage (Zhang et al., 2020). On the other hand, cuticular waxes can form slippery films or crystals that impede the attachment of pests to the plant surface (Mitchell et al., 2016). Additionally, wax deposition has also been reported as a plant response to abiotic stress, since it is considered a tolerance trait to drought and heat (Dhanyalakshmi et al., 2019). Moya–Raygoza (2016) recorded less damage in the teosinte *Zea perennis* (Hitchc.) and *Zea mays* ssp. *parviglumis* (H. H. Iltis & Doebley) caused by *S. frugiperda* (Lepidoptera: Noctuidae) when a higher density of trichomes was found in the upper leaf blade.

Another plant mechanism of response to the attack of arthropod herbivores is reducing plant palatability through the production of compounds that are toxic or harmful to the intestinal function of arthropods (War et al., 2012). The compounds that have been studied the most are alkaloids, benzoxazinoids, and terpenoids (Mithöfer and Boland, 2012). Benzoxazinoids are a group of compounds that play a defensive role in maize plants against arthropod herbivores (Qi et al., 2018). Yan et al. (1999) demonstrated that DIMBOA (2,4-dihydroxy-7-metoxy-1,4-benzoxazin-3-one), a benzoxazinoid metabolite, is toxic to the Asian corn borer [*Ostrinia furnacalis* (Guenée); Lepidoptera: Crambidae]. Maize plants have been shown to have a defense system against the attack of *Spodoptera littoralis* and *S. frugiperda*. This defense system consists of the accumulation and emission of HDMBOA (2-hydroxy-4, 7-dimetoxy-1, 4-benzoxazin-3-one), which is a toxic compound for these both herbivorous arthropods (Glauser et al., 2011).

The mechanisms of tolerance to biotic stress by herbivory are related to the alteration of physiological processes such as photosynthetic activity and growth, the plant phenological stage, and the use of nutrients stored in the plant (Mitchell et al., 2016). Partial defoliation due to arthropod herbivory can lead to an increase in the photosynthetic rate in the remaining tissues (Redondo–Gómez, 2013; Mitchell et al., 2016). A higher supply of leaf or root cytokinins due to partial defoliation can increase CO_2_ fixation and nutrient transport and assimilation (Redondo–Gómez, 2013). Additionally, there may be increases in the production of the Rubisco enzyme and the chlorophyll content of the remaining leaf tissue, which could improve the photosynthetic rate (Turnbull et al., 2007; Redondo–Gómez, 2013). Mitchell et al. (2016) mentions the activation of dormant buds after damage to reproductive or vegetative meristems as a tolerance mechanism that allows the plant to recover from the attack of arthropod herbivores. In maize plants, the delay in the allocation of resources (carbohydrates and nutrients) can generate tolerance to the attack of the western corn rootworm (*Diabrotica virgifera virgifera* LeConte; Coleoptera: Chrysomelidae; Robert et al., 2015).

## Responses of Maize Plants to the Combination Between Drought or High Temperatures and Arthropod Herbivory

Climate models predict a continuous increase in temperature and greater rainfall variability, with increments in drought and extreme temperature periods in the future (Gutbrodt et al., 2012; Grinnan et al., 2013). Global climate change is expected to affect the interaction between arthropods and plants through alterations in the physiology, behavior, and life cycle parameters of arthropod pests, and morphological, physiological, and biochemical changes in host plants (Cornelissen, 2011). Increases in temperatures are highly correlated with changes in phenology, distributions, abundance, and interactions between species and help to improve arthropod survival during extreme environments (Grinnan et al., 2013; Bansal, 2015). On the other hand, water shortage episodes can generate negative or positive effects on herbivore populations, depending on the intensity and frequency of stress (Bansal, 2015). Drought periods are associated with high temperatures that can accelerate the metabolism of insects, increasing their growth rate, consumption, and development (Neven, 2000; Bansal, 2015). Loss of turgor due to severe drought conditions could limit the availability of nitrogen-containing compounds for arthropods that feed on sap, reducing their performance (Gely et al., 2020).

Increases in temperature and drought can cause more frequent and severe outbreaks of herbivorous arthropod populations (Grinnan et al., 2013). These environmental stresses can cause a reduction in plant defense compounds against arthropods. The drop in these compounds increases the availability of nitrogen and proteins which can lead to more palatable food for some herbivorous arthropods (Gutbrodt et al., 2011). The synergistic combination between environmental stresses (drought or heat) and the increase in arthropod pest populations could lead to a reduction in plant yield (Grinnan et al., 2013). On the other hand, water deficit can cause an increase in the concentrations of secondary metabolites in some plant species, limiting the performance of herbivorous arthropods (Nguyen et al., 2018). Exposure to multiple environmental and biotic stress factors (arthropods) can have interactive effects on secondary metabolites such as volatile or non-volatile compounds since they share metabolic pathways with hormones such as jasmonic acid and salicylic acid (Scott et al., 2019). Copolovici et al. (2014) observed an interactive response between drought and herbivory mediated by methyl salicylate (derived from salicylic acid), resulting in a faster emission of compounds such as β-ocimene and 4,8-dimethyl-1,3,7-nonatriene (DMNT) in drought-stressed plants.

The accumulation of non-protein amino acids such as 5-hydroxynorvaline under herbivory and drought has been observed in maize (Yan et al., 2015). These types of compounds are defense metabolites to protect the plant against arthropod herbivores due to their poor incorporation during protein synthesis and/or the inhibition of biosynthetic pathways in primary metabolism (Huang et al., 2011). The accumulation of 5-hydroxynorvaline has been reported during the herbivory of corn leaf aphids (*Rhopalosiphum maidis*) and beet armyworm (*Spodoptera exigua*; Yan et al., 2015). These same authors observed a greater accumulation of non-protein amino acid 5-hydroxynorvaline under water deficit in plants under the herbivory of the corn leaf aphid and beet armyworm. The inhibition mainly of the aphid’s reproduction indicates that this secondary metabolite can have a defense function in maize plants (Yan et al., 2015). On the other hand, the accumulation of antimicrobial compounds of low molecular weight (terpenoid phytoalexins) has also been reported in response to the combination of biotic and abiotic factors in plants (Schmelz et al., 2014). In maize, two new families of terpenoid phytoalexins, zealexins, and kauralexins have been identified. These families show high antifungal and anti-feedant activity for insects; however, little is known about their role in responses to abiotic factors (Block et al., 2019). The accumulation of phytoalexins, mainly in roots, may be mediated by ABA signaling (Yang et al., 2012). Vaughan et al. (2015) showed a greater accumulation of zealexins and kauralexins (non-volatile terpenoid compounds) in maize roots exposed to herbivory by *Diabrotica balteata* under water shortage. The production of zealexins and kauralexins in plants is a defense response to biotic stresses that affects directly the growth and reproduction of arthropod pests in maize crops (Block et al., 2019). Finally, the benzoxazinoids production has also been reported as one of the main metabolites of direct defense in maize plants under drought, insect herbivory, or their combination. These stress factors favor the accumulation of benzoxazinoids through the systemic signaling of ABA (Vaughan et al., 2015).

## Conclusion and Future Perspectives

**FIGURE 2:**
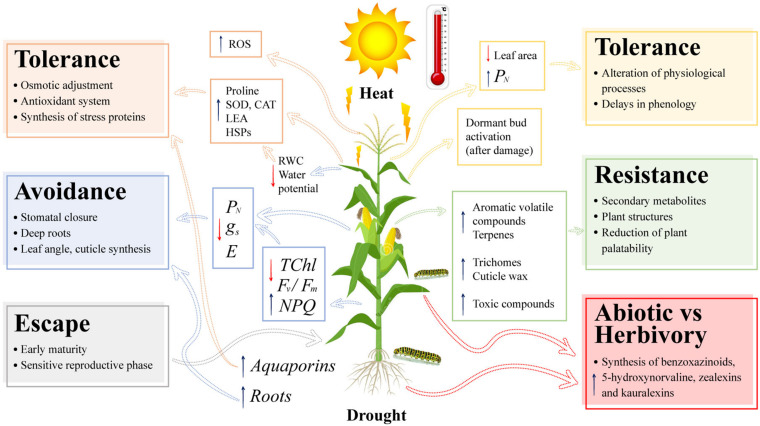
Concept model of the morphological, physiological, biochemical, and molecular responses of maize plants (*Zea mays* L.) to the impact of the combination of drought, heat stress, and arthropod herbivory. *P*_*N*_, net photosynthesis rate; g*_*s*_*, stomatal conductance; *E*, plant transpiration; TChl, total chlorophyll content; F_v_/F_m_, maximum quantum efficiency of PSII; NPQ, non-photochemical quenching; ROS, reactive oxygen species; SOD, superoxide dismutase; CAT, catalase; LEA, late embryogenesis abundant proteins; HSPs, heat shock proteins; and RWC, relative water content. Dotted arrows highlight responses and tolerance mechanisms of plants to single (drought, heat, or herbivory arthropod) or combined stresses. Blue or red arrows represent increase or decrease of plant responses at physiological, biochemical and molecular levels, respectively.

Plants face the combination of different types of abiotic and biotic stresses. The physiological, biochemical, and molecular responses of maize plants to drought, high daytime temperatures, and the effect on some arthropod herbivores are summarized in a concept model (Figure 2). Maize plants show a wide range of responses to drought and heat that generate alterations in plant growth and morphology. Plants have developed different strategies to survive under hot and dry environments. Escape, avoidance, and tolerance are the mechanisms that work under the combination of these two environmental stresses (Figure 2).

Early maturation, changes in stomatal regulation, root system, and plant architecture constitute the responses related to escape and avoidance. A decrease in photosynthesis associated with stomatal closure, low plant water content, low photochemical activity of PSII, and degradation of photosynthetic pigments are responses to the combination of drought and heat, with the reproductive phase being the most sensitive to this combination. Tolerance is regulated by biochemical and molecular mechanisms such as compatible osmolyte synthesis, increased enzymatic activity, accumulation of plant growth regulators, increased expression of water transporting and stress proteins, and TFs (Figure 2). The presence of arthropod herbivores generates resistance responses in maize plants, such as synthesis of volatile and non-volatile compounds, structural traits such as trichomes and cuticle waxes, and the production of toxic compounds such as benzoxazinoids. Tolerance traits to arthropod herbivory are related to an improvement in the photosynthetic efficiency and regulation of the plant’s phenological cycle. Finally, the combination of environmental stresses and arthropod herbivory in maize plants can increase the production of secondary metabolites such as volatile and non-volatile compounds, improving the defense response of maize to arthropod pest infestations (Figure 2).

There is still a lack of knowledge about the combination of environmental factors such as drought and high temperatures on arthropod pest behavior and its effect on the physiology of the maize crop. Therefore, it is necessary to design and carry out experiments that can reveal different aspects of the combinations between stresses. To do this, it is required to adjust protocols for the exposure of plants to each type of stress so that they consider the same combination of factors that occur under field conditions. It is also recommended to evaluate agronomic strategies to improve tolerance to the combination of abiotic and biotic stresses. Some of these strategies may include soil management, irrigation practices, selection of the most appropriate crop varieties, and the application of genome and transgenic editing tools and technologies.

## Author Contributions

CC-A and HR-D wrote the manuscript. GL-M and AR-G revised and critically evaluated the manuscript. All authors contributed to the article and approved the submitted version.

## Conflict of Interest

The authors declare that the research was conducted in the absence of any commercial or financial relationships that could be construed as a potential conflict of interest.
